# Supplementary material for: The Effect of Ketoanalogues on Chronic Kidney Disease Deterioration: A Meta-Analysis
Source: Nutrients. 2019 Apr 26;11(5):957. doi: 10.3390/nu11050957 (PMC6566830; doi:10.3390/nu11050957)
Supplement: Supplementary file 1 [file nutrients-11-00957-s001.pdf]

Supplementary Table 1. The list of search terms on PubMed and Embase.

PubMed	Embase
(((((((((((((renal[All Fields] AND ("physiology"[Subheading] OR "physiology"[All Fields] OR "function"[All Fields] OR "physiology"[MeSH Terms] OR "function"[All Fields])) OR ("erbb receptors"[MeSH Terms] OR ("erbb"[All Fields] AND "receptors"[All Fields]) OR "erbb receptors"[All Fields] OR "egfr"[All Fields])) OR (estimated[All Fields] AND ("glomerular filtration rate"[MeSH Terms] OR ("glomerular"[All Fields] AND "filtration"[All Fields] AND "rate"[All Fields]) OR "glomerular filtration rate"[All Fields]))) OR ("electrolytes"[MeSH Terms] OR "electrolytes"[All Fields] OR "electrolyte"[All Fields])) OR ("calcium"[MeSH Terms] OR "calcium"[All Fields])) AND ("phosphorus, dietary"[MeSH Terms] OR ("phosphorus"[All Fields] AND "dietary"[All Fields]) OR "dietary phosphorus"[All Fields] OR "phosphorus"[All Fields] OR "phosphorus"[MeSH Terms])) OR ("creatinine"[MeSH Terms] OR "creatinine"[All Fields])) OR BUN[All Fields]) OR ("blood urea nitrogen"[MeSH Terms] OR ("blood"[All Fields] AND "urea"[All Fields] AND "nitrogen"[All Fields]) OR "blood urea nitrogen"[All Fields])) OR (("urea"[MeSH Terms] OR "urea"[All Fields]) AND ("nitrogen"[MeSH Terms] OR "nitrogen"[All Fields]) AND ("blood"[Subheading] OR "blood"[All Fields] OR "blood"[MeSH Terms]) AND level[All Fields])) OR ("albumins"[MeSH Terms] OR "albumins"[All Fields] OR "albumin"[All Fields])) OR ("nutritional status"[MeSH Terms] OR ("nutritional"[All Fields] AND "status"[All Fields]) OR "nutritional status"[All Fields])) OR ("cholesterol"[MeSH Terms] OR "cholesterol"[All Fields])) AND (((ketoanalogs[All Fields] OR ("keto acids"[MeSH Terms] OR ("keto"[All Fields]	('chronic kidney failure'/exp OR 'chronic kidney disease' OR 'chronic kidney disorder' OR 'chronic kidney failure' OR 'chronic kidney insufficiency' OR 'chronic nephropathy' OR 'chronic renal disease' OR 'chronic renal failure' OR 'chronic renal insufficiency' OR 'kidney chronic failure' OR 'kidney disease, chronic' OR 'kidney failure, chronic' OR 'kidney function, chronic disease' OR 'renal insufficiency, chronic' OR 'kidney injury'/exp OR 'acute renal injury' OR 'chronic kidney injury' OR 'chronic renal injury' OR 'kidney cortex lesion' OR 'kidney damage' OR 'kidney injury' OR 'kidney lesion' OR 'kidney trauma' OR 'renal damage' OR 'renal injury' OR 'renal lesion' OR 'renal trauma' OR 'trauma, kidney' OR 'trauma, renal' OR 'chronic kidney disease-mineral and bone disorder'/exp OR 'ckd-mbd' OR 'ckd-mineral and bone disorder' OR 'chronic kidney disease-mineral and bone disorder') AND ('α ketoacids' OR ketoacids OR ketoacid OR ketosterile OR ketoanalogs) AND ('kidney function'/exp OR 'function, kidney' OR 'kidney function' OR 'kidney physiology' OR 'renal function' OR 'estimated glomerular filtration rate'/exp OR 'egfr (estimated glomerular filtration rate)' OR 'estimated gfr' OR 'estimated glomerular filtration rate' OR 'estimated glomerulofiltration rate' OR 'estimated glomerulus filtration rate' OR 'egfr'/exp OR 'creatinine'/exp OR '1 methylglycocyamidine' OR '1 methylhydantoin 1 imide' OR '2 imino 1 methyl 4 imidazolinone' OR 'creatinin' OR 'creatinine' OR 'creatinine hydrochloride' OR 'kreatinine' OR 'methylglycocyamimine' OR 'urea nitrogen blood level'/exp OR 'bun' OR 'blood urea nitrogen' OR 'plasma urea nitrogen' OR 'serum bun' OR 'serum urea nitrogen' OR 'urea nitrogen blood level' OR 'nutritional status'/exp OR 'nutrition state' OR 'nutrition status' OR 'nutritional state'

AND "acids"[All Fields]) OR "keto acids"[All Fields] OR "ketoacids"[All Fields])) OR alpha-ketoacid[All Fields]) OR (alpha-keto[All Fields] AND ("acids"[MeSH Terms] OR "acids"[All Fields])))) AND (("kidney failure, chronic"[MeSH Terms] OR ("kidney"[All Fields] AND "failure"[All Fields] AND "chronic"[All Fields]) OR "chronic kidney failure"[All Fields] OR ("chronic"[All Fields] AND "kidney"[All Fields] AND "failure"[All Fields])) OR ("renal insufficiency, chronic"[MeSH Terms] OR ("renal"[All Fields] AND "insufficiency"[All Fields] AND "chronic"[All Fields]) OR "chronic renal insufficiency"[All Fields] OR ("chronic"[All Fields] AND "kidney"[All Fields] AND "disease"[All Fields]) OR "chronic kidney disease"[All Fields]))	OR 'nutritional status' OR 'albumin'/exp OR 'albumen' OR 'albumin' OR 'albumin secretion' OR 'albumin variant' OR 'liquid albumin' OR 'cholesterol'/exp OR 'electrolyte'/exp OR 'calcium'/exp OR 'phosphorous acid'/exp OR 'orthophosphite' OR 'phosphorous acid' OR 'phosphorous acids' OR phosphorous)
